# Assessment of nutritional status using anthropometric index among older adult and elderly population in India

**DOI:** 10.1038/s41598-023-39167-6

**Published:** 2023-08-10

**Authors:** Junaid Khan, Aparajita Chattopadhyay, Subhojit Shaw

**Affiliations:** Department of Population and Development, International Institute for Population Sciences, Deonar, Mumbai, 400088 India

**Keywords:** Geriatrics, Health services, Nutrition, Public health, Quality of life

## Abstract

Malnutrition poses a significant risk to the older population globally, highlighting the critical role of nutrition in healthy aging. In this study, the aim is to estimate the prevalence of malnutrition among older adults aged 45–59 years and the elderly population aged 60 years and above based on their socioeconomic and demographic characteristics. Furthermore, the study examines the risk factors within a multivariate framework. A sample of 59,073 individuals aged 45 years and above from the Longitudinal Aging Study in India (LASI), Wave 1 survey constitutes the study population. This study adopts a cross-sectional design. Bivariate cross-tabulation analysis and multivariate logistic regression analysis are employed to understand the prevalence and determinants of nutritional status. About 25% of males and 37% of females below the age of 60 years are overweight (including obese), while among those aged 60 years and above, 28% of males and 25% of females are underweight. The elderly male population carries a comparatively higher burden of underweight (28%) prevalence than the females (25%) in the same age group. Overall, the urban population is less likely to be underweight [AOR: 0.41, C.I 0.38–0.43] and more likely to be overweight [AOR: 2.41, C.I 2.32–2.52]. Older adults from low economic and social strata are more likely to be underweight. In terms of bio-physical factors, individuals aged 60 years and above with infections to endemic diseases [AOR: 1.24; p-value < 0.01] and those with edentulism [AOR: 1.29; p-value < 0.01] are more likely to be underweight. As evident from the study, nutritional status among older adults is a complicated manifestation of multiple risk factors and requires potential nutritional intervention. Initiating a routine screening program at the grassroots level can effectively identify older adults and the elderly in India, facilitating the provision of nutritional care.

## Introduction

The share of older population is growing globally and it is expected to rise by 300% in Asia and Latin America in the next 30 years^1,2^. According to the United Nations (UN), it is estimated that the older population of India will rise to 19.1% by 2050^3^. The upsurge in the older population is the result of decline in fertility rates and improvement in the life expectancy since the 1950s^4^. In the past 40 years, aging has taken a center stage in discussions, starting with the World Assembly on Ageing in Vienna in 1983, aiming towards the equal development and improvement of their health and wellbeing^5^. In January 1999, India passed the country's first-ever "National Policy on Older Persons". In 2010, the Government of India started the National Program for Health Care of the Elderly, dedicated to providing comprehensive health care to the older persons^6^. In India, with the advancement in medical science, an improved healthcare system and better living standards have shown possibility of staying healthy and living longer lives^7^. As life expectancy has improved during the last few decades in India, nutrition has a significant role in healthy aging especially when 60 + years population is likely to reach 340 million by 2050^6,8^.

Malnutrition is a condition that refers to a deficit or excess of various nutrients. Studies have established that as individuals grow older, their energy intake decreases, and anorexia is the most common cause of malnutrition among older individuals^9^. Adequate diet and proper nutrition are important health determinants in the older population. Notably, appropriate nutrition has the potential to prevent and delay certain chronic diseases that affect the older adults^10,11^. As individuals age, loss of bone density is a common condition that could raise the risk of osteoporosis. The other age-related alteration is sarcopenia, which causes loss of lean muscle mass and results in increased body fat^12^. With the progression of aging, the sense of smell diminishes, making food less appealing to them, and reduced saliva production contributes to constipation and other digestive problems^12^. All of these health issues change eating patterns and decrease nutrient availability and absorption, leading to nutritional deficiencies and various health problems. The nutritional deficiency among older adults has been identified within the three domains of wasting, which results from inadequate diet, cachexia, caused due to catabolism and severe chronic diseases and sarcopenia, the muscle inactivity illness over time which generally occurs with progressive age^13^. Therefore, involuntary weight loss, along with other chronic diseases and mental retardation, poses a challenge to healthy aging. In India, studies on nutritional status among older adults are sporadic^14^. Mathew et al. 2016 had conducted a study in the urban area in Coimbatore of South India and found 19% of the population are malnourished and 24% are at risk^15^. Another study by Lahiri et al., 2015 found that women are more malnourished (59%) than the males in West Bengal^16^. Studies conducted across different regions of India have shown that multi-morbidity and poor nutritional status are associated with increasing age and it is common across poor communities^17,18^.

Low and Middle income countries are experiencing an increase in overweight and obesity for demographic and epidemiological transition^19^. Globally, overweight and obesity were projected to be responsible for 3.4 million deaths worldwide in 2010^20^. At this juncture, women are the most critical subgroup to be affected by nutritional transition (underweight to overweight)^21^. Maternal underweight is linked to a higher chance of having small gestational period and low-birth-weight babies, while overweight is linked to a higher risk of congenital abnormalities in babies^22^. The rising prevalence of obesity has resulted in chronic sickness among adults, such as cardiovascular disease, diabetes, musculoskeletal diseases, and various cancers^23,24^.

With the progression of age, the aetiology of age-related disorders like low-grade inflammation emerges. Studies have shown that the gut microbiota plays a key role in inflammation, driven by nutrient excess or over nutrition^25^. The nutrient composition, quantity of meals, timing and rhythmicity all play a significant impact on the gut microbiota and metabolism, contributing to the maintenance of a low level of inflammation, which gets increased by over nutrition (metaflammation). The existing reference states that metabolic abnormalities caused by a high-fat diet (overnutrition) and obesity can lead to type-2 diabetes^26,27^. Diabetes increases immune system activity and as a result, pro-inflammatory cytokine releases, which can contribute to brain neuroinflammation, cognitive decline and Alzheimer's-like dementia^28,29^. Moreover, studies have highlighted the problem of overweight and obesity with the increased risk of functional and mobility limitations among the older individuals^30,31^.

In the recent past, nutritional frailty has gained the attention of many researchers^32–34^. Nutritional frailty is more prevalent among the older adults, resulting in rapid weight loss, loss of muscle mass and strength, exhaustion, low grip strength, leaving the individual susceptible to physiological impairment^35,36^. Sarcopenia is one of the most common problems at this age, where the loss of skeletal muscle mass leads to functional disability and mortality^37^. A study found a significant association between protein imbalance and body mass change among older adults^38^. On the similar ground, Isanejad and collegues 2015 has found the severity of frailty syndrome and sarcopenia in association with insufficient nutrition intake among Finnish older women^39^. Weight loss generally precedes the development of cognitive decline and Alzheimer's disease^40^. Empirical studies have shown evidence that the Mediterranean diet, exclusively based on fruits, seafood, vegetables, and olive oil, helps in moderating cognitive health and Alzheimer's disease^41,42^. With deteriorating immunodeficiency, older adults are at a higher risk of infectious and inflammatory disorders with longer recovery times and higher fatality rates. Several studies have demonstrated a decrease in nutrient absorption (specifically Vitamin B6, Vitamin E, Zinc) in individuals over the age of 60 years, leading to impaired immune system functionality and diminished resistance to infections^43–46^.

The association of nutritional health and aging has been linked with biological, psychological, sociological and environmental factors. A complex pathway proposed by Kane and colleagues in 2009 explains the role of socio-demographic, psychological factors like cognitive health, food preferences and biomedical factors like chronic diseases, motor function, drugs on nutritional status among older persons^14,47^. In a community setting, social environment plays an important role in changing dietary patterns. When an elderly starts living alone, they experience loss of appetite accompanied with chewing problem and denial. Similarly, due to mobility restriction with co-morbidities, meal availability and accessibility becomes a major challenge. As a result, to address the nutrient shortage at this age, family support is essential.

In India, majority of nutritional program are focused on child, adolescent and maternal health. The magnitude of malnutrition among older adults is under- reported and few studies are conducted in the domain of nutritional status of the older adults^14,48^. The study of the nutritional status of older adults in India is a crucial area of research as this population is vulnerable to malnutrition. The Nutritional needs of older adults are different from those of younger adults. Poor nutritional health is a prevalent problem among older adults in India due to poverty, lack of education, and limited healthcare or nutrition program access^14,48^. Malnutrition in older adults can lead to various health problems, including weakened immune systems, increased risk of infections, reduced muscle mass, impaired wound healing, and increased mortality rates^49,50^. Furthermore, older adults' nutritional status can have significant implications for their cognitive and physical functioning, cognitive impairment, leading to a decline in quality of life^51,52^. Hence, studying the nutritional status of older adults in India is essential for identifying and addressing the problem of malnutrition among them. The findings of due research can help interventions to promote healthy aging and improve the quality of life of older adults in India.

## Materials and methods

### Data

**Figure 1:**
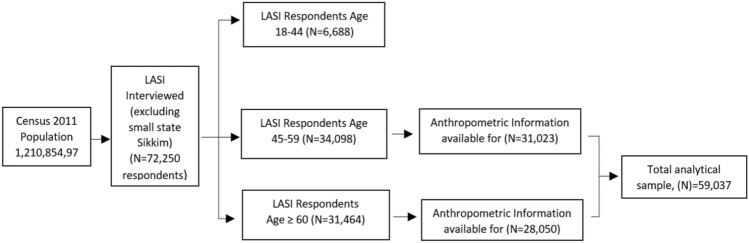
Diagrammatic flow chart shows the analytical sample of the study.

This study is based on Longitudinal Aging Study in India (LASI), Wave 1 data. The survey was conducted during 2017 to 2018 and collected information on individuals’ physical, social, and cognitive health across all states and union territories (UTs) in India. The pan India survey included men and women aged 45 and above, with a sample of 72,250 respondents. A multistage stratified area probability cluster sampling was used, with a three-stage design for rural areas and a four-stage design for urban areas^53^. The data is publicly available, and thus, no ethical review committee permission was required for this study. We have considered two age groups in the study; the older adults aged 45–59 years and elderly aged 60 years and above. Of the total sample, we have considered the individuals aged 45 and above excluding spouses less than 45 years, consisting of 65,562 respondents. Out of the 65,562 respondents, the anthropometric information is complete for 59,073 respondents. The sample of older adults in the age group of 45–59 years consists of 31,023 individuals whereas the sample of elderly aged 60 years and above consists of 28,050 individuals. Figure 1 shows the flow chart of the analytical sample. Throughout the manuscript, following the LASI definition, the population aged 45–59 years is referred to as “older adults,” and the population aged 60 years and above is referred to as “the elderly”.

### Outcome variable

To measure nutritional status among older adults, this study utilized the Body Mass Index (BMI), one of the most distinctive measures recommended by the World Health Organization (WHO). BMI is defined as weight (kg) divided by height squared (m^2^). The classification of BMI follows as: below 18.5 as “underweight”, 18.5–24.9 as “normal”, 25–29.9 as “overweight”, 30–34.9 as “obese class I”, 35–39.9 as “obese class II,” and over 40 “obese class III”, indicating a risk to comorbidity^54^. The study participants were divided into three groups: underweight, normal, and overweight including obese. BMIs less than 18.5 were classified as underweight, 18.5–24.9 as normal, and 25 and above as overweight. LASI identifies BMI as an essential indicator to define the nutritional status of older adults. BMI is widely used as an anthropometric measure of body composition and a metric to identify nutritional status^55^. It helps identify different levels of health disease risk. On the other hand, the LASI survey data does not provide any other clinical information on nutrition. Consequently, we have exclusively used the BMI indicator to define the different forms of nutritional status among older adults in India.

### Exposure variables

In this study, we controlled various socio-demographic, economic, bio-physical and behavioral factors to determine the nutritional status among older adults in India. The factors include gender (male/female), place of residence (rural/urban), marital status (married/widow/others), living arrangement (living alone/living with spouse and/or others/living with spouse and children/living with children and others/ living with others only), educational level (no schooling/less than 5 years completed/5–9 years completed/10 or more years completed), social-class (scheduled caste/scheduled tribe/other backward class-OBC/general), household size (1–2 persons/3–4 persons/5 + persons), economic status in terms of monthly per capita expenditure (MPCE) (poorest/poorer/middle/richer/richest), food availability constraint (no constraint/low constraint/severe constraint), infection with any endemic diseases (no/yes), any mental health issues (no/yes), chewing capacity (no/partial or complete edentulism), tobacco consumption (no/yes), alcohol consumption (no/yes) and physical activity status (no/yes).

Besides considering the social, demographic and economic factors, we also considered the health and health behaviour related factors (infection of endemic disease, mental health issues, edentulism, tobacco consumption, alcohol consumption and physical activity) and its possible influence on malnutrition among older adults and elderly in India. LASI collects the specific information on endemic disease infection (water borne, vector-borne and other infectious disease like- tuberculosis and diarrhoea) prior to 2 years of the survey.

### Statistical analysis

The variables considered in the study are described using descriptive statistics. Box plot and multiple bar diagram are used to examine the range of BMI and the prevalence of different forms of nutritional status. Furthermore, bivariate cross-tabulation is used to estimate the prevalence of underweight and overweight/obese across the sub-populations. Additionally, three different logistic regression models are employed to determine the effect of selected socio-demographic, economic, bio-physical and behavioral factors on the outcome variable. Step-wise logistic regression models are estimated. Model-1 includes demographic variables; Model-2 includes demographic and economic variables; Model-3 includes demographic, economic and health variables; Model-4 includes demographic, economic, health and health behaviour variables. Model-4 is considered the final model and the corresponding results are tabulated in the main text. The extended tables are shown in the Supplementary Material. The multicollinearity between the exposure variables were checked and we did not find any VIF value beyond 2.

### Ethical approval

As the study is based on secondary data available in public domain for research; no ethical approval was required from any institutional review board (IRB).

## Results

Table 1 shows the description of the study population. Of the total 31,023 older adults, 39% belong to the 45–49 years age group. Forty-five percent of the older adults are male, 64% belong to rural areas, 85% are married and almost one-fifth of them belongs to the poorest wealth quintile. On the other hand, of the total elderly, 48% are males and 52% are females. The rural–urban divide reveals that 66% of the elderly population resides in rural areas. Additionally, only 64% of the elderly are married, while 33% are widowed. Around 20% of the elderly belongs to the poorest MPCE quintile.

**Table 1:** Descriptive statistics of the study population, India, LASI Wave 1, 2017–18.

Variables	45–59 years (Older adults)		60 + years (Elderly)	
	N	Distribution (%)	N	Distribution (%)
**Age in years**				
45–49	11,985	38.63	NA	NA
50–59	19,038	61.37	NA	NA
60–70	NA	NA	19,211	68.49
70 +	NA	NA	8839	31.51
**Sex**				
Male	13,834	44.59	13,509	48.16
Female	17,189	55.41	14,541	51.84
**Residence**				
Rural	19,991	64.44	18,689	66.63
Urban	11,032	35.56	9361	33.37
**Marital status**				
Married	26,285	84.73	17,952	64
Widowed	3557	11.47	9389	33.47
Others	1180	3.8	709	2.53
**Living arrangement**				
Living alone	641	2.07	1459	5.2
Living with spouse and/or others	3547	11.43	5499	19.6
Living with spouse and children	22,149	71.4	12,251	43.68
Living with children and others	3702	11.93	7417	26.44
Living with others only	984	3.17	1424	5.08
**Education**				
No schooling	12,771	41.17	14,984	53.42
Less than 5 years complete	3428	11.05	3449	12.3
5–9 years complete	8104	26.12	5425	19.34
10 or more years complete	6720	21.66	4192	14.94
**Caste**				
Scheduled caste	5367	17.3	4579	16.32
Scheduled tribe	5746	18.52	4634	16.52
Other backward class (OBC)	11,601	37.39	10,663	38.01
None of them	8309	26.78	8174	29.14
**HH size**				
1–2	8331	27.17	9532	34.49
3–4	18,162	59.23	13,986	50.61
5 +	4173	13.61	4116	14.89
**MPCE quintile**				
Poorest	5933	19.12	5722	20.4
Poorer	6131	19.76	5797	20.67
Middle	6163	19.87	5769	20.57
Richer	6384	20.58	5528	19.71
Richest	6412	20.67	5234	18.66
**Food availability constraint**				
No constraint	11,462	36.95	10,326	36.81
Low constraint	17,677	56.98	15,808	56.36
Severe constraint	1884	6.07	1916	6.83
**Any endemic disease**				
No	23,161	74.66	20,736	73.94
Yes	7861	25.34	7308	26.06
**Any mental health issues**				
No	30,447	98.17	27,364	97.59
Yes	566	1.83	677	2.41
**Edentulism**				
No	13,759	44.36	4478	15.97
Partial or complete	17,260	55.64	23,565	84.03
**Tobacco consumption**				
No	21,403	69.08	19,013	67.86
Yes	9582	30.92	9003	32.14
**Alcohol consumption**				
No	25,204	81.32	23,163	82.67
Yes	5790	18.68	4857	17.33
**Physical activity**				
No	8927	28.8	12,691	45.29
Yes	22,068	71.2	15,333	54.71
**Total**	31,023	100	28,050	100

### Nutritional status of older adults and elderly

**Figure 2:**
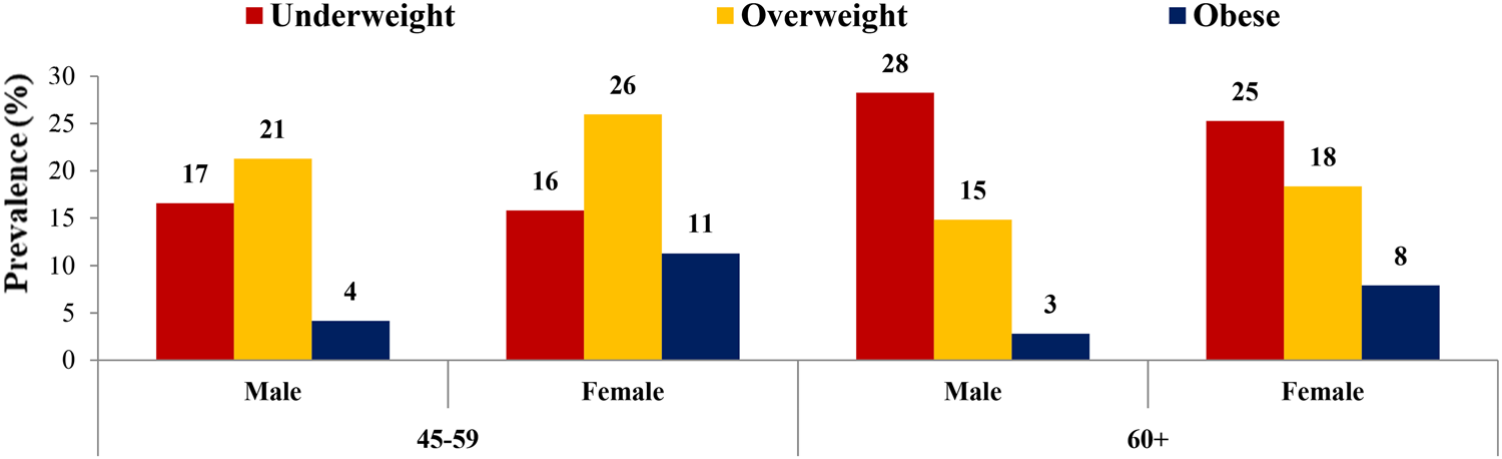
Prevalence of underweight, overweight and obese by age and sex among older adults & elderly, India, LASI Wave 1, 2017–18.

The prevalence of nutritional status of older adults and the elderly is shown in Fig. 2. Though the prevalence of underweight is almost equal among male and female older adults, females aged 45–59 years have a higher burden of overweight (26%) and obesity (11%) compared to males of the same age. The proportion of obese males is lower (4%) than the females (11%) in the 45–59 years of age group. Similar to the pattern of older adults, elderly females demonstrate higher overweight and obesity prevalence among them than the prevalence among elderly males. On the other hand, males aged 60 years and above carry a comparatively higher burden of underweight (28%) than females (25%).

**Figure 3:**
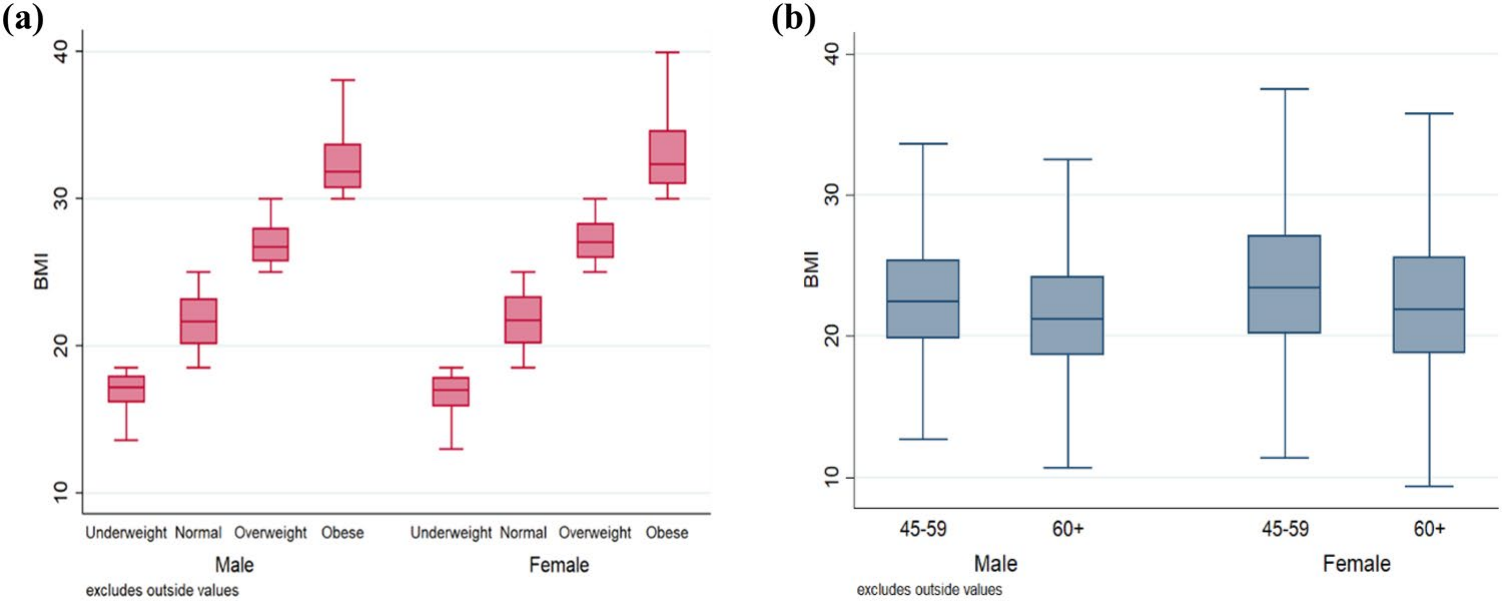
Box Plot showing the BMI range (**a**) within group heterogeneity by nutritional status; (**b**) within-group heterogeneity by age group and sex in the study population, India, LASI Wave 1, 2017–18.

Figure 3a shows the range of BMI by sex and nutritional status. The boxes are almost uniform for males and females in nutritional status except for obese males and females, means the BMI values are comparatively more dispersed above the median value among obese females than obese males. The age-sex pattern of BMI is shown in Fig. 3b. It is observed that the BMI of female older adults and elderly is more scattered compared the males. The median BMI is observed high among the older adult females than all other sub-groups.

### Prevalence of underweight and overweight (includes obese) by selected characteristics

Table 2 shows the age-sex pattern of prevalence of underweight and overweight (overweight includes obese) by background characteristics of the study population. In the rural areas, the prevalence of underweight is higher than the urban areas; male (34%) and female (31%) elderly carry almost similar burden of underweight. In contrast, the prevalence of overweight is high in the urban areas irrespective of age and sex and the prevalence of overweight is as high as 58% among female older adults.

**Table 2:** Prevalence of underweight and overweight among male and female older adults and elderly by selected background characteristics, India, LASI Wave 1, 2017–18.

Background characteristics	Underweight				Overweight (includes obese)			
	Male		Female		Male		Female	
	Older adult	Elderly	Older adult	Elderly	Older adult	Elderly	Older adult	Elderly
**Residence**								
Rural	20.46	33.72	19.79	31.21	18.14	13.02	27.25	17.70
Urban	7.98	13.04	7.26	11.54	41.43	30.35	58.44	45.89
**Marital status**								
Married	15.93	26.38	14.33	21.66	26.07	18.75	38.19	29.14
Widowed	27.98	36.45	17.86	28.22	14.10	12.94	35.69	23.89
Others	23.36	35.57	35.86	28.01	17.79	10.75	24.83	23.22
**Living arrangement**								
Living alone	22.64	34.25	17.55	30.00	12.75	14.07	30.69	20.82
Living with spouse and/or others	15.72	27.58	15.54	21.91	24.11	17.89	38.63	26.79
Living with spouse and children	16.00	25.95	14.02	21.69	26.37	19.22	38.21	30.46
Living with children and others	24.71	34.64	17.29	26.99	15.81	13.64	36.88	25.19
Living with others only	24.17	40.76	37.86	32.11	19.14	7.55	21.99	19.88
**Education**								
No schooling	24.78	38.81	20.25	30.63	14.21	9.34	27.68	18.41
Less than 5 years complete	23.49	31.29	14.92	16.01	16.86	14.71	35.63	32.65
5–9 years complete	15.43	25.15	7.43	11.84	26.07	18.34	49.74	47.55
10 or more years complete	7.39	11.10	8.61	2.79	38.40	33.26	62.32	65.39
**Caste**								
Scheduled caste	20.57	35.81	18.76	31.84	18.05	11.54	29.31	18.75
Scheduled tribe	31.05	43.97	29.54	37.94	12.25	7.22	18.98	8.13
Other backward class (OBC)	13.97	27.36	14.63	24.45	28.21	17.95	39.46	28.64
None of them	12.74	20.17	10.51	18.16	30.96	24.08	46.15	33.01
**HH size**								
1–2	17.49	28.26	16.56	26.57	23.88	17.31	37.62	23.07
3–4	16.06	28.98	15.72	24.91	26.61	17.53	36.21	27.75
5 +	17.72	26.70	16.05	26.26	21.81	18.15	33.67	23.43
**MPCE quintile**								
Poorest	20.62	36.01	21.87	34.68	18.39	11.42	27.84	16.28
Poorer	20.71	34.69	17.05	28.68	19.45	15.29	32.85	20.64
Middle	18.19	26.96	15.94	26.53	21.01	18.40	37.09	25.17
Richer	13.54	23.79	16.13	18.09	30.04	18.97	37.70	34.69
Richest	8.96	17.29	7.13	14.49	39.43	25.57	52.12	38.91
**Food availability constraint**								
No constraint	16.96	28.68	16.06	26.21	22.60	17.76	38.36	25.60
Low constraint	15.14	25.80	14.68	23.27	28.61	18.71	38.23	27.88
Severe constraint	25.96	43.50	22.84	34.26	13.88	9.20	23.94	18.26
**Any endemic disease**								
No	14.74	25.93	14.89	23.51	27.72	19.03	38.50	28.72
Yes	21.63	34.43	18.22	29.74	19.00	13.82	33.74	19.91
Any mental health issues*								
No	16.60	28.38	15.90	25.02	25.42	17.63	36.89	26.35
Yes	15.03	23.63	10.05	34.00	25.38	16.47	53.67	22.79
**Edentulism**								
No	14.57	23.31	15.84	21.70	27.32	20.93	36.14	30.86
Partial or complete	18.36	29.31	15.74	25.94	23.69	16.90	38.04	25.38
**Tobacco consumption**								
No	10.82	20.95	13.64	22.06	33.94	23.92	39.99	28.54
Yes	21.90	35.42	27.53	38.33	17.53	11.39	22.06	16.89
**Alcohol consumption**								
No	14.19	27.16	15.29	24.82	28.25	18.56	37.85	26.64
Yes	21.68	31.03	33.31	41.83	19.38	15.13	14.87	11.74
**Physical activity**								
No	15.75	28.95	15.00	26.56	28.43	18.11	39.13	25.29
Yes	16.92	27.65	16.02	24.33	24.15	17.13	36.64	26.94

*The difference is not statistically significant (chi-square value) for male older adults, male and female elderly.

Widowed men reflect higher prevalence of underweight (37%) than widowed women. Compared to married elderly males (overweight prevalence-19%), married older adult males (overweight prevalence-26%) reflect higher prevalence and likewise, the prevalence of overweight is comparatively higher among married older adult females than their counter part males.

The prevalence of underweight is predominantly higher among the individuals who stay alone. It is also observed that among 60 + years male (underweight prevalence-34%) and female (underweight prevalence-30%) who are living alone are underweight compared to the older adult males and females. The estimated prevalence by educational status of older adults shows that those who are not educated, the prevalence of underweight is found higher for both the sexes while the prevalence of overweight is observed higher among the highly educated males. In the case of 60 + years population with no schooling, the prevalence of underweight is 39% among males and 31% among females. In contrast, the prevalence of overweight is comparatively higher among better educated female older adults (62%) as well as among female elderly (65%). The prevalence of underweight and overweight shows a difference among different social classes of India. Elderly males and females from the scheduled tribe and scheduled caste classes carry higher burden of underweight than all other social classes. The observed prevalence of underweight among ST elderly males is found 44% and 38% among ST elderly females. On the other hand, the prevalence of overweight is higher among the individuals from the upper class.

Those living with more than five members in the household, experience highest prevalence of underweight (18%). Females from the lowest MPCE quintile demonstrate a higher prevalence of underweight among them compared to females from higher MPCE quintiles. Older adults from the richest MPCE quintile demonstrate a higher burden of overweight, with a prevalence of 39% among males and 52% among females. Similarly, elderly persons from the lowest MPCE quintile demonstrate higher prevalence of underweight problem. Similarly, individuals facing constraints in food availability in household, experience a higher burden of underweight e.g., elderly males facing severe food availability constraints demonstrate a prevalence of underweight at 44%.

Elderly as well as older adult males and females with mental health issues do not exhibit a higher burden of undernutrition compared to those without mental health issues, except for female older adults. Female older adults with mental health issues demonstrate a 54% prevalence of overweight. Both elderly and older adults with partial/complete edentulism experience a higher burden of underweight, with approximately 30% of elderly males and 26% of elderly females being underweight. It is observed that individuals who consume tobacco carry higher burden of underweight prevalence than those who do no consume tobacco. Almost two-fifth of the elderly females who consume tobacco are underweight (underweight prevalence-38%). Alcohol consumption also demonstrates a higher burden of underweight. The prevalence of underweight is as high as 42% among the female elderly who consume alcohol. Both males and females in both the age groups who are not involved in physical activity carry higher burden of overweight than those who are involved in physical activity. It is observed that almost two-fifth of the older adult females are overweight.

### Determinants of underweight and overweight (includes obese)

#### Socio-demographic factors

Table 3 provides a detailed understanding of the determinants of underweight and overweight (overweight also includes obese) among older adults as well as among the elderly in India. Results show that females are less likely to be underweight, while overweight and obesity phenomenon is more likely to occur among the females. An elderly female is 20% less likely (AOR: 0.80; p-value < 0.01) to be underweight than a male elderly. The urban elderly and older adults are both less likely to be underweight with AOR 0.41 and 0.40 respectively compared to their rural counterparts. Widowed and other non-married individuals are more likely to be underweight than those who are married across all the age groups. Elderly who lives with spouse and/or others are 25% more likely (AOR: 1.25; p-value < 0.01) to be underweight and those elderly (with no spouse) who live with children and others are 19% more likely to be underweight (AOR: 1.19, p-value < 0.05). Individuals with higher educational qualification are less likely to be underweight than individuals who have never attended school. On the contrary, the higher educated elderly as well as the older adults are more likely to be overweight compared to their lesser educated counterparts. The pattern of malnutrition prevalence across social strata has revealed that people in higher social strata are less likely to be underweight. The upper-caste individuals have the lowest odds (AOR: 0.81; p-value < 0.01) of being underweight. Individuals who are not married are less prone to be overweight (AOR: 0.76, p-value < 0.01).

**Table 3:** Multivariate logistic estimation for underweight and overweight (overweight includes obese) in India, LASI Wave 1, 2017–2018.

Background Characteristics	Underweight			Overweight (includes obese)		
	Total	60 + years	45–59 years	Total	60 + years	45–59 years
**Sex**						
Male®						
Female	0.82*** (0.78–0.87)	0.80*** (0.74–0.86)	0.98 (0.90–1.06)	2.00*** (1.91–2.10)	2.14*** (1.98–2.31)	1.74*** (1.63–1.86)
**Residence**						
Rural®						
Urban	0.41*** (0.38–0.43)	0.41*** (0.38–0.44)	0.40*** (0.37–0.44)	2.41*** (2.32–2.52)	2.35*** (2.20–2.50)	2.48*** (2.35–2.63)
**Marital status**						
Married®						
Widowed	1.76*** (1.40–2.22)	1.50** (1.03–2.18)	1.52*** (1.12–2.07)	0.76*** (0.64–0.89)	1.08 (0.74–1.57)	0.87 (0.72–1.06)
Others	1.72*** (1.33–2.24)	1.50* (0.99–2.27)	1.75*** (1.24–2.46)	0.76*** (0.63–0.93)	1.07 (0.71–1.63)	0.73*** (0.58–0.92)
**Living arrangement**						
Living alone®						
Living with spouse and/or others	1.40** (1.08–1.80)	1.25 (0.84–1.85)	1.23 (0.85–1.78)	1.05 (0.87–1.27)	1.43* (0.96–2.11)	1.14 (0.89–1.47)
Living with spouse and children	1.28* (0.99–1.65)	1.29 (0.87–1.92)	1.20 (0.83–1.74)	1.06 (0.88–1.29)	1.33 (0.90–1.98)	1.07 (0.84–1.37)
Living with children and others	1.06 (0.94–1.21)	1.19** (1.02–1.38)	0.91 (0.70–1.17)	1.13* (1.00–1.28)	1.00 (0.85–1.18)	1.27** (1.03–1.56)
Living with others only	1.22** (1.05–1.43)	1.20* (0.99–1.44)	1.33* (0.99–1.79)	0.95 (0.81–1.11)	0.92 (0.76–1.13)	0.96 (0.75–1.23)
**Education**						
No schooling®						
Less than 5 years complete	0.75*** (0.70–0.80)	0.70*** (0.64–0.77)	0.84*** (0.75–0.93)	1.35*** (1.27–1.45)	1.50*** (1.36–1.65)	1.22*** (1.11–1.34)
5–9 years complete	0.55*** (0.52–0.59)	0.60*** (0.55–0.65)	0.55*** (0.50–0.60)	1.82*** (1.73–1.92)	1.83*** (1.68–1.99)	1.71*** (1.59–1.83)
10 or more years complete	0.36*** (0.33–0.40)	0.37*** (0.32–0.42)	0.39*** (0.35–0.45)	2.20*** (2.07–2.34)	2.34*** (2.13–2.58)	1.98*** (1.83–2.14)
**Caste**						
Scheduled caste®						
Scheduled tribe	0.97 (0.91–1.04)	0.97 (0.88–1.07)	0.98 (0.88–1.08)	0.70*** (0.65–0.75)	0.73*** (0.65–0.83)	0.68*** (0.62–0.75)
Other backward class (OBC)	0.94** (0.89–1.00)	0.95 (0.88–1.03)	0.91** (0.83–0.99)	1.08** (1.02–1.14)	1.14*** (1.04–1.26)	1.05 (0.98–1.14)
None of them	0.83*** (0.77–0.89)	0.81*** (0.74–0.89)	0.81*** (0.73–0.91)	1.21*** (1.14–1.29)	1.24*** (1.12–1.36)	1.25*** (1.16–1.36)
**HH Size**						
1–2®						
3–4	0.88*** (0.83–0.94)	0.86*** (0.79–0.94)	0.88** (0.80–0.97)	1.18*** (1.12–1.25)	1.20*** (1.10–1.31)	1.20*** (1.12–1.29)
5 +	0.76*** (0.70–0.82)	0.75*** (0.67–0.84)	0.71*** (0.63–0.81)	1.39*** (1.30–1.50)	1.40*** (1.25–1.57)	1.48*** (1.34–1.63)
**MPCE quintile**						
Poorest®						
Poorer	0.85*** (0.80–0.90)	0.89*** (0.81–0.97)	0.79*** (0.72–0.87)	1.25*** (1.17–1.34)	1.28*** (1.16–1.42)	1.24*** (1.14–1.36)
Middle	0.67*** (0.63–0.72)	0.68*** (0.62–0.74)	0.67*** (0.61–0.74)	1.48*** (1.38–1.58)	1.53*** (1.38–1.69)	1.45*** (1.33–1.59)
Richer	0.53*** (0.50–0.57)	0.54*** (0.49–0.60)	0.53*** (0.47–0.59)	1.90*** (1.78–2.03)	2.01*** (1.82–2.23)	1.83*** (1.68–2.00)
Richest	0.39*** (0.36–0.43)	0.42*** (0.38–0.47)	0.36*** (0.32–0.41)	2.53*** (2.36–2.70)	2.44*** (2.20–2.71)	2.60*** (2.38–2.85)
**Food availability constraint**						
No constraint®						
Low constraint	0.96 (0.92–1.01)	0.95 (0.89–1.01)	0.97 (0.90–1.04)	1.03 (0.99–1.08)	1.00 (0.94–1.07)	1.07 (1.01–1.13)
Severe constraint	1.35*** (1.24–1.47)	1.35*** (1.20–1.51)	1.39*** (1.23–1.58)	0.84*** (0.76–0.92)	0.87* (0.76–1.00)	0.79*** (0.70–0.90)
**Any endemic disease**						
No®						
Yes	1.28*** (1.22–1.34)	1.24*** (1.16–1.33)	1.34*** (1.24–1.44)	0.86*** (0.82–0.90)	0.82*** (0.76–0.88)	0.89*** (0.83–0.94)
**Any mental health issues**						
No®						
Yes	0.89 (0.76–1.04)	0.92 (0.75–1.12)	0.81 (0.62–1.06)	1.21*** (1.06–1.37)	1.10 (0.91–1.32)	1.36*** (1.13–1.63)
**Edentulism**						
No®						
Partial or complete	1.39*** (1.32–1.47)	1.29*** (1.18–1.40)	1.22*** (1.14–1.31)	0.82*** (0.78–0.85)	0.77*** (0.71–0.83)	0.95** (0.90–1.00)
**Tobacco consumption**						
No®						
Yes	1.72*** (1.64–1.81)	1.72*** (1.61–1.84)	1.75*** (1.63–1.89)	0.61*** (0.58–0.64)	0.62*** (0.58–0.67)	0.59*** (0.55–0.63)
**Alcohol consumption**						
No®						
Yes	1.15*** (1.08–1.22)	1.10** (1.02–1.20)	1.26*** (1.15–1.38)	0.96 (0.90–1.02)	0.96 (0.87–1.05)	0.93* (0.86–1.01)
**Physical activity**						
No®						
Yes	0.90*** (0.85–0.94)	0.91*** (0.86–0.97)	1.00 (0.92–1.07)	0.95** (0.91–0.99)	0.90*** (0.85–0.96)	0.91*** (0.86–0.96)

®: Reference Category, ***p < 0.01, **p < 0.05, *p < 0.1.

#### Economic factors

Elderly living in households with higher household size are less likely to be underweight. For example, elderly from the household with ‘3–4’ members are 14% less likely to be underweight and similarly elderly from the household with ‘5 + ’ household size is 25% less likely to be underweight compared to those elderly from the household size of ‘1–2’. Older adults as well as the elderly from the poorest MPCE quintile are more likely to be underweight than those from the richer MPCE class. The estimated odds associated with underweight for different MPCE classes demonstrate that the odds show a gradual decrease over the higher MPCE classes and individuals from the richest MPCE class carry the lowest odds of being underweight. In the overall 45 + years population, it is found that individuals from the richest MPCE quintile are more likely to be overweight (AOR 2.53; p-value < 0.01) and this pattern is similar in both the age groups. On the other hand, persons who experience severe household food insecurity in availability of food are 39% more likely to be underweight (AOR: 1.39; p-value < 0.01).

#### Bio-physical factors

In general, individuals who have suffered endemic diseases are at a higher risk of being underweight and elderly aged 60 + years with endemic disease infection are 24% more likely to be underweight (AOR 1.24; p-value < 0.01). Presence of any mental illness does not show any statistically significant association with underweight. But individuals with any kind of mental illnesses are more likely to be overweight. Edentulism shows a strong relation with both underweight and overweight, i.e. individuals aged 60 and above with edentulism (partial or complete) are 29% (AOR 1.29; p-value < 0.01) more likely to be underweight; while, older adults with edentulism are 22% (AOR: 1.22; p-value < 0.01) more likely to be underweight. Individuals who consume tobacco carry substantially high odds to be underweight. The elderly individuals who consume tobacco (smoke or smokeless or both) are 72% (AOR: 1.72, p-value < 0.01) more likely to be underweight while older adults who consume tobacco are 75% (AOR: 1.75, p-value < 0.01) more likely to be underweight. Individuals who consume alcohol are more likely to become underweight. While those who are engaged in some physical activities like engagement in daily chores to vigorous activities like running, swimming, gym, heavy lifting etc. are less likely to become underweight or overweight across all ages.

## Discussions

Nutritional status of the older adults and elderly remains a public health challenge in India. In this context, this study provides a detailed understanding of the nutritional status of the older adults aged 45–59 years and the population aged 60 years and above using Longitudinal Ageing Survey of India, wave1. With the advancement of medical science, there has been an increase in the share of older population attributed to rising life expectancy, changing social and family structures in India, that poses the question of older population care^56^. Issues related to malnutrition among the elderly have significantly increased scholarly attention in recent years for varying reasons^57–60^. India has shown an improvement in terms of child mortality and life expectancy and with increasing life expectancy the old-age dependency ratio has shown an increase. Although India has shown progress in terms of creating better healthcare infrastructure, facilitating improved healthcare, it is still grappling with chronic diseases, co-morbidities, and socio-economic disparities.

Adequate nutrition plays an essential role in maintaining overall health. Malnutrition among older adults is a manifestation of a variety of factors, and appropriate policy interventions are needed to combat against the public health burden of malnutrition among older adults and elderly^61,62^. The problem of underweight can be observed among the rural population. On the other hand, the problem of overweight is more dominated among the urban population. This could be attributable to the differences in the socioeconomic position, education, food availability, health care accessibility, sanitation etc.^63^. A study conducted by Abud et al. found that rural population in Ethiopia are significantly more malnourished (Mini Nutritional Assessment > 17) with (AOR = 2.08; 95% CI: 1.25–3.45)^57^. Another study indicated that 62 percent of Bangladesh's older adults are in danger of malnutrition^64^. This brings attention to the region-based health routine screening and nutritional program interventions, especially in the rural areas.

A crucial finding of this study is that females are more overweight than the males across all the ages. This condition has also been well observed in other studies of India, where females living in urban areas and with better economic condition demonstrate a higher prevalence of overweight and obesity^65,66^. This could be associated with increased women employment and improved socioeconomic position that results in sedentary lifestyle. Further, with increasing age, the metabolism of the body decreases and therefore a minimal or even a normal diet may cause weight gain^67,68^. Another study by Sinha and colleagues in 2018 highlighted the prevalence of overweight and obesity among post-menopausal women in India^69^. This study shows that the widows and unmarried individuals are more underweight while the prevalence of overweight is more among the married individuals. This indicates the loneliness experienced by elderly individuals, which exacerbates nutritional deficiencies. Despite the fact that the exact mechanism by which marriage gives health benefits is unknown, research has demonstrated that married older adults demonstrate better health and live longer^70^. This study informs that older adults who are not living with their spouse are at more risk of being underweight than those who live with their spouse. In contrast, a handful of studies show that adults are more likely to outlive their loved ones and friends with the progression of age, making them lonelier. Furthermore, the social constructs and home confinement lead to the risk of unnoticed falls or the inability to secure food supplies^71^.

Educational attainment plays a vital role as a predictor of nutritional status among the older adults. Previous studies have shown that with better health knowledge, the chances of being underweight reduces^61,72^. This study also shows that the higher educated elderly and older adults are less likely to be underweight and are more likely to be overweight. A systematic review by Kwan and colleagues in 2018 suggested that poverty is positively associated with low levels of education, low income, and difficulty in meeting the minimal nutritional demands^73^. Among the different social classes in India, the elderly as well as the older adult males and females from the scheduled caste (SC) and scheduled tribe (ST) class carry a higher burden and likelihood of underweight^59,74^.

In the present study, older adults aged 60 years and above who belong to the richest MPCE quintile has the lowest chance of being underweight; whereas the chance of being overweight is higher among them. These findings are supported by a study that suggests the income level of the caregivers enhances nutrition among older adults, while older adults who face abuse tend to skip meals and experience a deterioration in their health^59^. Another study found that food availability and affordability prevent elderly malnutrition^60^. The ever-changing family structure has been well documented in several studies in India^75–77^. The trend in change of family structure from joint to nuclear homes raises concerns about older adults' food intake or nutritional health. The findings are inconsistent with existing studies conducted in Thailand and South Africa that revealed an inverse association between increased household size and food quantity due to higher food expenditure and, as a result, inferior dietary quantity/quality^78,79^. The status of living alone among older adults can pose challenges related to self-care, cooking, and proper nutrition, which may increase the risk of being underweight. Additionally, a person's nutritional health is influenced by their financial stability and well-being. The other side of the scenario can be explained as being connected to consuming a diet that is higher in fat content, which can lead to obesity-related issues^80^.

Infection with endemic diseases substantially affects the nutritional health of older adults. Although the causal relationship between nutritional status and infection is bidirectional, infections themselves contribute to malnutrition both in children and adults^81^. Gastrointestinal infections that result in diarrhea, infections such as HIV/AIDS and tuberculosis, as well as other chronic infections that cause cachexia and anemia, along with parasitic infections in the intestines leading to anemia and nutritional deficiencies, are evidence indicating nutritional deficiencies caused by infections^81,82^. Stimulation of the immune response due to infection is very common, which causes a demand for metabolically derived anabolic energy, leading to a vicious cycle of adverse nutritional conditions and increased susceptibility to infection^83,84^.

The study shows that 60 + years males with infection to endemic diseases exhibit a significantly higher prevalence of underweight than males in the age group of 45–59 years. A similar pattern is observed among females. Whereas, the prevalence of overweight is observed to be lower among both male and females in both the age groups who had an infection with any of the endemic diseases (water borne, vector borne or other infectious diseases). The multivariable logistic estimation also shows a higher likelihood of underweight associated with the infection among both the older adult and elderly populations when adjusted for all other factors. Whereas, the likelihood of being overweight is found lower among those who had an infection. This indicates that infection with an endemic disease is a risk factor for underweight among both groups of individuals. This suggests that infection to endemic diseases generally leads to the problem of underweight than overweight.

In this study it is found that the 60 + years old women with mental health issues carry a higher prevalence of underweight. When controlled for all other covariates, we do not observe any statistically significant association between mental health issues and the problem of underweight. However, older adults aged 45–59 years are more likely to be overweight when they have mental health issues. Mental health and nutritional status are both age dependent and deteriorate with increasing age. Though good nutrition and healthy eating habit play an important role in mental health, a good mental wellbeing also determines the nutritional status^85–87^. A set of studies found that depression leads to changes in eating behaviour among older individuals and the treatment of depression causes weight loss^88–90^. Another study describes that depression is potentially associated with low appetite and low food-intake with increasing risk of malnutrition among the elderly^91^.

In general, the prevalence of underweight is observed to be higher among those who are partially or completely edentate, except for females aged 45–59 years. In comparison to older adults, elderly individuals carry higher prevalence of underweight. In contrast, the prevalence of overweight is generally lower among the partially or completely edentate except for women in the 45–59 years age group. The specific estimates of adjusted odds ratio of edentulism show a consistent and statistically significant association with underweight. This study shows that edentulism among older persons is a risk factor of underweight. Edentulism is associated with the food-groups consumed and hard-to-chew foods are commonly avoided among the elderly^92^. Elderly individuals who wear dentures and have compromised masticating capacity often experience more chewing pain compared to those with natural teeth. This discomfort affects their food group choices, leading to a higher risk of malnutrition among edentulous older persons^92–94^. Though there are confounding factors that affects the nutritional status among the older population, but tooth loss leads to reduction in intake of fruits and vegetables causing nutritional disturbance among them^95,96^.

The study also indicates that the prevalence of underweight is consistently high among the older adults as well as among the elderly population who consume tobacco. Notably, the prevalence of underweight is quite high among elderly persons who smoke compared to older adult smokers of both sexes. It is evident that consumption of tobacco is more of a risk factor which causes underweight among older adults as well as among the elderly. According to a previous study, cigarette smoking is a risk factor for undernutrition, and individuals who smoke have a higher likelihood of experiencing malnourishment^97^. A study also found that smoking negatively affects the eating habit among elderly reducing taste and smell, and anorectic effects of nicotine lower the appetite leading to a loss in body weight^98^. Like tobacco consumption, consumption of alcohol is also associated with malnutrition among elderly and it is a life-style risk factor of malnutrition^97^. Prevalence of malnutrition is higher among the alcohol consumers as alcohol consumption leads to impaired liver function affecting the protein metabolism in the body^99^. Alcohol consumption is also associated with mineral and vitamin deficiency which directly affects the nutritional status among the elderly^100^.

According to a previous study physical activity and nutrient intake both play important roles in maintaining good health throughout life, and neither of these two factors alone can help achieve optimal health unless they are combined^101^. Low cardiovascular fitness and the presence of chronic health conditions are often linked to physical inactivity, which is also a significant factor contributing to adult mortality^102^. Although nutritional status among the elderly is the manifestation of multiple factors and a complex interplay of different factors, but physical activity is associated with sarcopenia^103,104^.

## Limitations

The study has several limitations. Firstly, the cross-sectional design only captures a snapshot of data at a specific point in time, and thus failing to explore the causal relationships or bring understanding of the temporal nature of the variables under investigation. Additionally, the reliance on self-reported data in cross-sectional surveys like LASI may introduce recall bias, as participants may not accurately recall or report certain information. Furthermore, cross-sectional studies are unable to account for changes or variations that may occur over time, such as seasonal effects or fluctuations in participants' nutritional health status. Another limitation is the selection bias, as certain groups of individuals may be more likely to participate in the survey, leading to a non-representative sample of the population. Lastly, cross-sectional research may not capture the complexity of longitudinal trends or developmental processes, limiting the ability to understand long-term patterns and changes. Moreover, the population survey data does not capture the nuanced and multifactorial nature of malnutrition, which requires a more comprehensive assessment involving medical examinations, nutritional assessments, and laboratory tests. Therefore, while the LASI survey data can offer valuable insights, it is important to interpret the findings cautiously and consider these limitations in the context of studying malnutrition among older adults in India.

## Conclusion

Underweight and overweight are significant problems among older adults and the elderly in India. This study, the first of its kind to explain the problem of malnutrition using a nationally representative sample of the 45 years and older population and brings forth the evidence that malnutrition among the older population in India is quite substantial at the national level and across different population groups. Widowed individuals, those experiencing food insecurity, those who suffered from endemic disease, edentulism and poor health behaviour in terms of tobacco and alcohol consumption require attention to address the underweight problem among older adults. Female older adults aged 45–59 years are at a higher risk of being overweight. Urban population, individuals with mental disorders and sedentary life-style are at the risk of being overweight. Females across ages carry a higher burden of being overweight, and males in India experience a higher prevalence of underweight, especially above the age of 60 years.

It is critical to meet older individuals’ nutritional demands to ensure their overall health, and quality of life. As poverty, food availability, community where they live, chronic and mental disease and their way of living are important factors which determine the nutritional condition. Thus, more multidisciplinary research needs to be conducted to gather community-based nutrition knowledge and initiate a routine screening of older adults in the population. It is also necessary to develop appropriate preventive strategies during early adulthood to reduce the burden of underweight across different sub-populations, considering individualized nutritional needs and introducing physical examination of nutritional health. It is recommended to encourage the adult population to refrain from unhealthy behaviour, promote oral health and mental wellbeing among them to address nutritional problems. Improving the situation by addressing malnutrition among older adults and the elderly in India might prove challenging as their nutritional status reflects past experiences and practices. Therefore, the program in India should be robust from adulthood to effectively reduce the burden of malnutrition during the later phases of life.

### Supplementary Information

Supplementary Tables.

## Data Availability

This study is based upon Longitudinal Aging Study in India (LASI), Wave 1 data which is publicly available through International Institute for Population Sciences data repository and could be accessed upon a data request. The request form is available at https://www.iipsindia.ac.in/content/lasi-publications.
