# Supplementary material for: Assessment of nutritional status using anthropometric index among older adult and elderly population in India
Source: Sci Rep. 2023 Aug 10;13:13015. doi: 10.1038/s41598-023-39167-6 (PMC10415320; doi:10.1038/s41598-023-39167-6)
Supplement: Supplementary file 1 — Supplementary Tables. [file 41598_2023_39167_MOESM1_ESM.docx]

Table1. Step wise logistic regression for underweight among older adults (45+) in India, LASI, 2017-18

| **Background Characteristics** | **Underweight** | | | |
| --- | --- | --- | --- | --- |
|  | **Total** | | | |
|  | Model-1 | Model-2 | Model-3 | Model-4 |
| **Sex** |  |  |  |  |
| Male |  |  |  |  |
| Female | 0.617*** | 0.635*** | 0.639*** | 0.824*** |
| **Residence** |  |  |  |  |
| Rural |  |  |  |  |
| Urban | 0.421*** | 0.386*** | 0.389*** | 0.406*** |
| **Marital Status** |  |  |  |  |
| Married |  |  |  |  |
| Widowed | 2.008*** | 1.900*** | 1.835*** | 1.762*** |
| Others | 1.786*** | 1.712*** | 1.724*** | 1.725*** |
| **Living arrangement** |  |  |  |  |
| Living alone |  |  |  |  |
| Living with spouse and/or others | 1.512*** | 1.442** | 1.423** | 1.396* |
| Living with spouse and children | 1.376** | 1.267 | 1.292* | 1.279 |
| Living with children and others | 1.092 | 1.054 | 1.065 | 1.064 |
| Living with others only | 1.296*** | 1.218* | 1.236** | 1.221* |
| **Education** |  |  |  |  |
| No schooling |  |  |  |  |
| Less than 5 years complete | 0.717*** | 0.754*** | 0.760*** | 0.751*** |
| 5-9 years complete | 0.477*** | 0.524*** | 0.536*** | 0.551*** |
| 10 or more years complete | 0.253*** | 0.314*** | 0.329*** | 0.363*** |
| **Caste** |  |  |  |  |
| Scheduled caste |  |  |  |  |
| Scheduled tribe | 1.009 | 0.986 | 0.984 | 0.971 |
| Other backward class (OBC) | 0.882*** | 0.918** | 0.911** | 0.942 |
| None of them | 0.729*** | 0.827*** | 0.813*** | 0.830*** |
| **HH Size** |  |  |  |  |
| 1-2 |  |  |  |  |
| 3-4 |  | 0.906** | 0.894*** | 0.882*** |
| 5+ |  | 0.801*** | 0.773*** | 0.759*** |
| **MPCE quintile** |  |  |  |  |
| Poorest |  |  |  |  |
| Poorer |  | 0.866*** | 0.859*** | 0.848*** |
| Middle |  | 0.686*** | 0.678*** | 0.674*** |
| Richer |  | 0.539*** | 0.535*** | 0.535*** |
| Richest |  | 0.392*** | 0.387*** | 0.393*** |
| **Food availability constraint** |  |  |  |  |
| No constraint |  |  |  |  |
| Low constraint |  | 0.996 | 0.967 | 0.962 |
| Severe constraint |  | 1.468*** | 1.395*** | 1.352*** |
| **Any endemic disease** |  |  |  |  |
| No |  |  |  |  |
| Yes |  |  | 1.298*** | 1.278*** |
| **Any mental health issues** |  |  |  |  |
| No |  |  |  |  |
| Yes |  |  | 0.908 | 0.888 |
| **Edentulism** |  |  |  |  |
| No |  |  |  |  |
| Partial or complete |  |  | 1.395*** | 1.393*** |
| **Tobacco consumption** |  |  |  |  |
| No |  |  |  |  |
| Yes |  |  |  | 1.724*** |
| **Alcohol consumption** |  |  |  |  |
| No |  |  |  |  |
| Yes |  |  |  | 1.145*** |
| **Physical activity** |  |  |  |  |
| No |  |  |  |  |
| Yes |  |  |  | 0.896*** |

**Model-1** includes demographic variables; **Model-2** includes demographic and economic variables; **Model-3** includes demographic, economic and health variables; **Model-4** includes demographic, economic, health and health behaviour variables.

Table2. Step wise logistic regression for underweight among older adults aged 60 and above in India, LASI, 2017-18

| **Background Characteristics** | **Underweight** | | | |
| --- | --- | --- | --- | --- |
|  | **60+** | | | |
|  | Model-1 | Model-2 | Model-3 | Model-4 |
| **Sex** |  |  |  |  |
| Male |  |  |  |  |
| Female | 0.635*** | 0.649*** | 0.647*** | 0.802*** |
| **Residence** |  |  |  |  |
| Rural |  |  |  |  |
| Urban | 0.420*** | 0.385*** | 0.390*** | 0.411*** |
| **Marital Status** |  |  |  |  |
| Married |  |  |  |  |
| Widowed | 1.530* | 1.509* | 1.530* | 1.495* |
| Others | 1.463 | 1.441 | 1.47 | 1.499 |
| **Living arrangement** |  |  |  |  |
| Living alone |  |  |  |  |
| Living with spouse and/or others | 1.234 | 1.24 | 1.26 | 1.248 |
| Living with spouse and children | 1.246 | 1.272 | 1.295 | 1.291 |
| Living with children and others | 1.156* | 1.193* | 1.191* | 1.185* |
| Living with others only | 1.231* | 1.221* | 1.225* | 1.196 |
| **Education** |  |  |  |  |
| No schooling |  |  |  |  |
| Less than 5 years complete | 0.678*** | 0.710*** | 0.715*** | 0.704*** |
| 5-9 years complete | 0.532*** | 0.575*** | 0.583*** | 0.600*** |
| 10 or more years complete | 0.265*** | 0.323*** | 0.332*** | 0.367*** |
| **Caste** |  |  |  |  |
| Scheduled caste |  |  |  |  |
| Scheduled tribe | 0.967 | 0.957 | 0.953 | 0.968 |
| Other backward class (OBC) | 0.888** | 0.925 | 0.920* | 0.953 |
| None of them | 0.697*** | 0.795*** | 0.791*** | 0.811*** |
| **HH Size** |  |  |  |  |
| 1-2 |  |  |  |  |
| 3-4 |  | 0.886** | 0.879** | 0.863*** |
| 5+ |  | 0.783*** | 0.767*** | 0.750*** |
| **MPCE quintile** |  |  |  |  |
| Poorest |  |  |  |  |
| Poorer |  | 0.900* | 0.893** | 0.886** |
| Middle |  | 0.680*** | 0.677*** | 0.676*** |
| Richer |  | 0.542*** | 0.540*** | 0.542*** |
| Richest |  | 0.416*** | 0.413*** | 0.424*** |
| **Food availability constraint** |  |  |  |  |
| No constraint |  |  |  |  |
| Low constraint |  | 0.972 | 0.952 | 0.952 |
| Severe constraint |  | 1.434*** | 1.382*** | 1.347*** |
| **Any endemic disease** |  |  |  |  |
| No |  |  |  |  |
| Yes |  |  | 1.263*** | 1.243*** |
| **Any mental health issues** |  |  |  |  |
| No |  |  |  |  |
| Yes |  |  | 0.932 | 0.915 |
| **Edentulism** |  |  |  |  |
| No |  |  |  |  |
| Partial or complete |  |  | 1.280*** | 1.287*** |
| **Tobacco consumption** |  |  |  |  |
| No |  |  |  |  |
| Yes |  |  |  | 1.721*** |
| **Alcohol consumption** |  |  |  |  |
| No |  |  |  |  |
| Yes |  |  |  | 1.103* |
| **Physical activity** |  |  |  |  |
| No |  |  |  |  |
| Yes |  |  |  | 0.910** |

**Model-1** includes demographic variables; **Model-2** includes demographic and economic variables; **Model-3** includes demographic, economic and health variables; **Model-4** includes demographic, economic, health and health behaviour variables.

Table3. Step wise logistic regression for underweight among older adults aged 45-59 in India, LASI, 2017-18

| **Background Characteristics** | **Underweight** | | | |
| --- | --- | --- | --- | --- |
|  | **45-59** | | | |
|  | Model1 | Model2 | Model3 | Model4 |
| **Sex** |  |  |  |  |
| Male |  |  |  |  |
| Female | 0.690*** | 0.715*** | 0.711*** | 0.977 |
| **Residence** |  |  |  |  |
| Rural |  |  |  |  |
| Urban | 0.414*** | 0.379*** | 0.384*** | 0.400*** |
| **Marital Status** |  |  |  |  |
| Married |  |  |  |  |
| Widowed | 1.729*** | 1.588** | 1.584** | 1.522** |
| Others | 1.865*** | 1.753** | 1.774*** | 1.748** |
| **Living arrangement** |  |  |  |  |
| Living alone |  |  |  |  |
| Living with spouse and/or others | 1.403 | 1.237 | 1.247 | 1.228 |
| Living with spouse and children | 1.439* | 1.181 | 1.202 | 1.204 |
| Living with children and others | 1.039 | 0.889 | 0.899 | 0.909 |
| Living with others only | 1.483** | 1.263 | 1.296 | 1.331 |
| **Education** |  |  |  |  |
| No schooling |  |  |  |  |
| Less than 5 years complete | 0.802*** | 0.842** | 0.847** | 0.836*** |
| 5-9 years complete | 0.475*** | 0.529*** | 0.536*** | 0.550*** |
| 10 or more years complete | 0.273*** | 0.344*** | 0.355*** | 0.394*** |
| **Caste** |  |  |  |  |
| Scheduled caste |  |  |  |  |
| Scheduled tribe | 1.065 | 1.028 | 1.022 | 0.977 |
| Other backward class (OBC) | 0.840*** | 0.876** | 0.874** | 0.905* |
| None of them | 0.708*** | 0.801*** | 0.795*** | 0.813*** |
| **HH Size** |  |  |  |  |
| 1-2 |  |  |  |  |
| 3-4 |  | 0.896* | 0.883* | 0.882* |
| 5+ |  | 0.737*** | 0.713*** | 0.714*** |
| **MPCE quintile** |  |  |  |  |
| Poorest |  |  |  |  |
| Poorer |  | 0.816*** | 0.810*** | 0.794*** |
| Middle |  | 0.686*** | 0.675*** | 0.670*** |
| Richer |  | 0.533*** | 0.527*** | 0.526*** |
| Richest |  | 0.364*** | 0.358*** | 0.364*** |
| **Food availability constraint** |  |  |  |  |
| No constraint |  |  |  |  |
| Low constraint |  | 1.023 | 0.99 | 0.966 |
| Severe constraint |  | 1.549*** | 1.453*** | 1.392*** |
| **Any endemic disease** |  |  |  |  |
| No |  |  |  |  |
| Yes |  |  | 1.361*** | 1.335*** |
| **Any mental health issues** |  |  |  |  |
| No |  |  |  |  |
| Yes |  |  | 0.831 | 0.812 |
| **Edentulism** |  |  |  |  |
| No |  |  |  |  |
| Partial or complete |  |  | 1.232*** | 1.222*** |
| **Tobacco consumption** |  |  |  |  |
| No |  |  |  |  |
| Yes |  |  |  | 1.754*** |
| **Alcohol consumption** |  |  |  |  |
| No |  |  |  |  |
| Yes |  |  |  | 1.256*** |
| **Physical activity** |  |  |  |  |
| No |  |  |  |  |
| Yes |  |  |  | 0.995 |

**Model-1** includes demographic variables; **Model-2** includes demographic and economic variables; **Model-3** includes demographic, economic and health variables; **Model-4** includes demographic, economic, health and health behaviour variables.

Table4. Step wise logistic regression for overweight or obesity among older adults (45+) in India, LASI, 2017-18

| **Background Characteristics** | **Overweight or Obese** | | | |
| --- | --- | --- | --- | --- |
|  | **Total** | | | |
|  | Model-1 | Model-2 | Model-3 | Model-4 |
| **Sex** |  |  |  |  |
| Male |  |  |  |  |
| Female | 2.407*** | 2.339*** | 2.334*** | 2.002*** |
| **Residence** |  |  |  |  |
| Rural |  |  |  |  |
| Urban | 2.288*** | 2.527*** | 2.512*** | 2.414*** |
| **Marital Status** |  |  |  |  |
| Married |  |  |  |  |
| Widowed | 0.699*** | 0.740*** | 0.759** | 0.757** |
| Others | 0.734** | 0.771** | 0.770** | 0.763** |
| **Living arrangement** |  |  |  |  |
| Living alone |  |  |  |  |
| Living with spouse and/or others | 0.999 | 1.059 | 1.07 | 1.05 |
| Living with spouse and children | 1.023 | 1.083 | 1.075 | 1.061 |
| Living with children and others | 1.130* | 1.147* | 1.143* | 1.132* |
| Living with others only | 0.924 | 0.963 | 0.953 | 0.949 |
| **Education** |  |  |  |  |
| No schooling |  |  |  |  |
| Less than 5 years complete | 1.405*** | 1.339*** | 1.333*** | 1.354*** |
| 5-9 years complete | 2.042*** | 1.870*** | 1.847*** | 1.825*** |
| 10 or more years complete | 2.976*** | 2.438*** | 2.366*** | 2.203*** |
| **Caste** |  |  |  |  |
| Scheduled caste |  |  |  |  |
| Scheduled tribe | 0.690*** | 0.692*** | 0.692*** | 0.698*** |
| Other backward class (OBC) | 1.131*** | 1.102** | 1.106*** | 1.078* |
| None of them | 1.364*** | 1.219*** | 1.236*** | 1.213*** |
| **HH Size** |  |  |  |  |
| 1-2 |  |  |  |  |
| 3-4 |  | 1.176*** | 1.185*** | 1.184*** |
| 5+ |  | 1.362*** | 1.394*** | 1.395*** |
| **MPCE quintile** |  |  |  |  |
| Poorest |  |  |  |  |
| Poorer |  | 1.233*** | 1.241*** | 1.251*** |
| Middle |  | 1.466*** | 1.475*** | 1.476*** |
| Richer |  | 1.891*** | 1.902*** | 1.898*** |
| Richest |  | 2.548*** | 2.572*** | 2.526*** |
| **Food availability constraint** |  |  |  |  |
| No constraint |  |  |  |  |
| Low constraint |  | 1.006 | 1.021 | 1.034 |
| Severe constraint |  | 0.793*** | 0.816*** | 0.837*** |
| **Any endemic disease** |  |  |  |  |
| No |  |  |  |  |
| Yes |  |  | 0.847*** | 0.860*** |
| **Any mental health issues** |  |  |  |  |
| No |  |  |  |  |
| Yes |  |  | 1.203** | 1.207** |
| **Edentulism** |  |  |  |  |
| No |  |  |  |  |
| Partial or complete |  |  | 0.818*** | 0.816*** |
| **Tobacco consumption** |  |  |  |  |
| No |  |  |  |  |
| Yes |  |  |  | 0.612*** |
| **Alcohol consumption** |  |  |  |  |
| No |  |  |  |  |
| Yes |  |  |  | 0.956 |
| **Physical activity** |  |  |  |  |
| No |  |  |  |  |
| Yes |  |  |  | 0.949* |

**Model-1** includes demographic variables; **Model-2** includes demographic and economic variables; **Model-3** includes demographic, economic and health variables; **Model-4** includes demographic, economic, health and health behaviour variables.

Table5. Step wise logistic regression for overweight or obesity among older adults aged 60 and above in India, LASI, 2017-18

| **Background Characteristics** | **Overweight or Obese** | | | |
| --- | --- | --- | --- | --- |
|  | **60+** | | | |
|  | Model1 | Model2 | Model3 | Model4 |
| **Sex** |  |  |  |  |
| Male |  |  |  |  |
| Female | 2.474*** | 2.413*** | 0.647*** | 2.141*** |
| **Residence** |  |  |  |  |
| Rural |  |  |  |  |
| Urban | 2.278*** | 2.495*** | 0.390*** | 2.348*** |
| **Marital Status** |  |  |  |  |
| Married |  |  |  |  |
| Widowed | 1.101 | 1.105 | 1.530* | 1.08 |
| Others | 1.1 | 1.121 | 1.47 | 1.072 |
| **Living arrangement** |  |  |  |  |
| Living alone |  |  |  |  |
| Living with spouse and/or others | 1.506* | 1.485* | 1.26 | 1.425 |
| Living with spouse and children | 1.468* | 1.383 | 1.295 | 1.33 |
| Living with children and others | 1.072 | 1.01 | 1.191* | 1.001 |
| Living with others only | 0.948 | 0.933 | 1.225* | 0.925 |
| **Education** |  |  |  |  |
| No schooling |  |  |  |  |
| Less than 5 years complete | 1.558*** | 1.488*** | 0.715*** | 1.497*** |
| 5-9 years complete | 2.025*** | 1.873*** | 0.583*** | 1.829*** |
| 10 or more years complete | 3.099*** | 2.583*** | 0.332*** | 2.343*** |
| **Caste** |  |  |  |  |
| Scheduled caste |  |  |  |  |
| Scheduled tribe | 0.740*** | 0.738*** | 0.953 | 0.734*** |
| Other backward class (OBC) | 1.205*** | 1.169*** | 0.920* | 1.145** |
| None of them | 1.419*** | 1.256*** | 0.791*** | 1.237*** |
| **HH Size** |  |  |  |  |
| 1-2 |  |  |  |  |
| 3-4 |  | 1.199*** | 0.879** | 1.204*** |
| 5+ |  | 1.379*** | 0.767*** | 1.400*** |
| **MPCE quintile** |  |  |  |  |
| Poorest |  |  |  |  |
| Poorer |  | 1.267*** | 0.893** | 1.281*** |
| Middle |  | 1.532*** | 0.677*** | 1.531*** |
| Richer |  | 2.024*** | 0.540*** | 2.013*** |
| Richest |  | 2.484*** | 0.413*** | 2.437*** |
| **Food availability constraint** |  |  |  |  |
| No constraint |  |  |  |  |
| Low constraint |  | 0.969 | 0.952 | 1.001 |
| Severe constraint |  | 0.824** | 1.382*** | 0.873 |
| **Any endemic disease** |  |  |  |  |
| No |  |  |  |  |
| Yes |  |  | 1.263*** | 0.820*** |
| **Any mental health issues** |  |  |  |  |
| No |  |  |  |  |
| Yes |  |  | 0.932 | 1.097 |
| **Edentulism** |  |  |  |  |
| No |  |  |  |  |
| Partial or complete |  |  | 1.280*** | 0.769*** |
| **Tobacco consumption** |  |  |  |  |
| No |  |  |  |  |
| Yes |  |  |  | 0.622*** |
| **Alcohol consumption** |  |  |  |  |
| No |  |  |  |  |
| Yes |  |  |  | 0.959 |
| **Physical activity** |  |  |  |  |
| No |  |  |  |  |
| Yes |  |  |  | 0.905** |

**Model-1** includes demographic variables; **Model-2** includes demographic and economic variables; **Model-3** includes demographic, economic and health variables; **Model-4** includes demographic, economic, health and health behaviour variables.

Table6. Step wise logistic regression for overweight or obesity among older adults aged 45-59 in India, LASI, 2017-18

| **Background Characteristics** | **Overweight or Obese** | | | |
| --- | --- | --- | --- | --- |
|  | **45-59** | | | |
|  | Model-1 | Model-2 | Model-3 | Model-4 |
| **Sex** |  |  |  |  |
| Male |  |  |  |  |
| Female | 2.170*** | 2.104*** | 2.111*** | 1.743*** |
| **Residence** |  |  |  |  |
| Rural |  |  |  |  |
| Urban | 2.328*** | 2.590*** | 2.573*** | 2.484*** |
| **Marital Status** |  |  |  |  |
| Married |  |  |  |  |
| Widowed | 0.802* | 0.865 | 0.865 | 0.873 |
| Others | 0.690** | 0.727** | 0.723** | 0.728** |
| **Living arrangement** |  |  |  |  |
| Living alone |  |  |  |  |
| Living with spouse and/or others | 1.024 | 1.159 | 1.158 | 1.143 |
| Living with spouse and children | 0.948 | 1.094 | 1.091 | 1.072 |
| Living with children and others | 1.142 | 1.290* | 1.292* | 1.266* |
| Living with others only | 0.877 | 0.992 | 0.982 | 0.961 |
| **Education** |  |  |  |  |
| No schooling |  |  |  |  |
| Less than 5 years complete | 1.253*** | 1.198*** | 1.196*** | 1.222*** |
| 5-9 years complete | 1.902*** | 1.733*** | 1.727*** | 1.708*** |
| 10 or more years complete | 2.664*** | 2.168*** | 2.144*** | 1.983*** |
| **Caste** |  |  |  |  |
| Scheduled caste |  |  |  |  |
| Scheduled tribe | 0.662*** | 0.665*** | 0.665*** | 0.680*** |
| Other backward class (OBC) | 1.112** | 1.084* | 1.083* | 1.055 |
| None of them | 1.408*** | 1.269*** | 1.271*** | 1.255*** |
| **HH Size** |  |  |  |  |
| 1-2 |  |  |  |  |
| 3-4 |  | 1.198*** | 1.203*** | 1.198*** |
| 5+ |  | 1.461*** | 1.483*** | 1.479*** |
| **MPCE quintile** |  |  |  |  |
| Poorest |  |  |  |  |
| Poorer |  | 1.222*** | 1.226*** | 1.244*** |
| Middle |  | 1.438*** | 1.443*** | 1.452*** |
| Richer |  | 1.817*** | 1.825*** | 1.830*** |
| Richest |  | 2.622*** | 2.636*** | 2.604*** |
| **Food availability constraint** |  |  |  |  |
| No constraint |  |  |  |  |
| Low constraint |  | 1.033 | 1.045 | 1.066* |
| Severe constraint |  | 0.754*** | 0.770*** | 0.795*** |
| **Any endemic disease** |  |  |  |  |
| No |  |  |  |  |
| Yes |  |  | 0.869*** | 0.886*** |
| **Any mental health issues** |  |  |  |  |
| No |  |  |  |  |
| Yes |  |  | 1.338** | 1.359** |
| **Edentulism** |  |  |  |  |
| No |  |  |  |  |
| Partial or complete |  |  | 0.941* | 0.948* |
| **Tobacco consumption** |  |  |  |  |
| No |  |  |  |  |
| Yes |  |  |  | 0.592*** |
| **Alcohol consumption** |  |  |  |  |
| No |  |  |  |  |
| Yes |  |  |  | 0.929 |
| **Physical activity** |  |  |  |  |
| No |  |  |  |  |
| Yes |  |  |  | 0.910** |

**Model-1** includes demographic variables; **Model-2** includes demographic and economic variables; **Model-3** includes demographic, economic and health variables; **Model-4** includes demographic, economic, health and health behaviour variables.
